# Supplementary material for: Plasma Levels of a Cleaved Form of Galectin-9 Are the Most Sensitive Biomarkers of Acquired Immune Deficiency Syndrome and Tuberculosis Coinfection
Source: Biomolecules. 2020 Oct 30;10(11):1495. doi: 10.3390/biom10111495 (PMC7693693; doi:10.3390/biom10111495)
Supplement: Supplementary file 1 [file biomolecules-10-01495-s001.zip › biomolecules-943414-SI/Biomolecules(proof)_Suppli Figs_2020.10.29.pptx]

## Slide 1
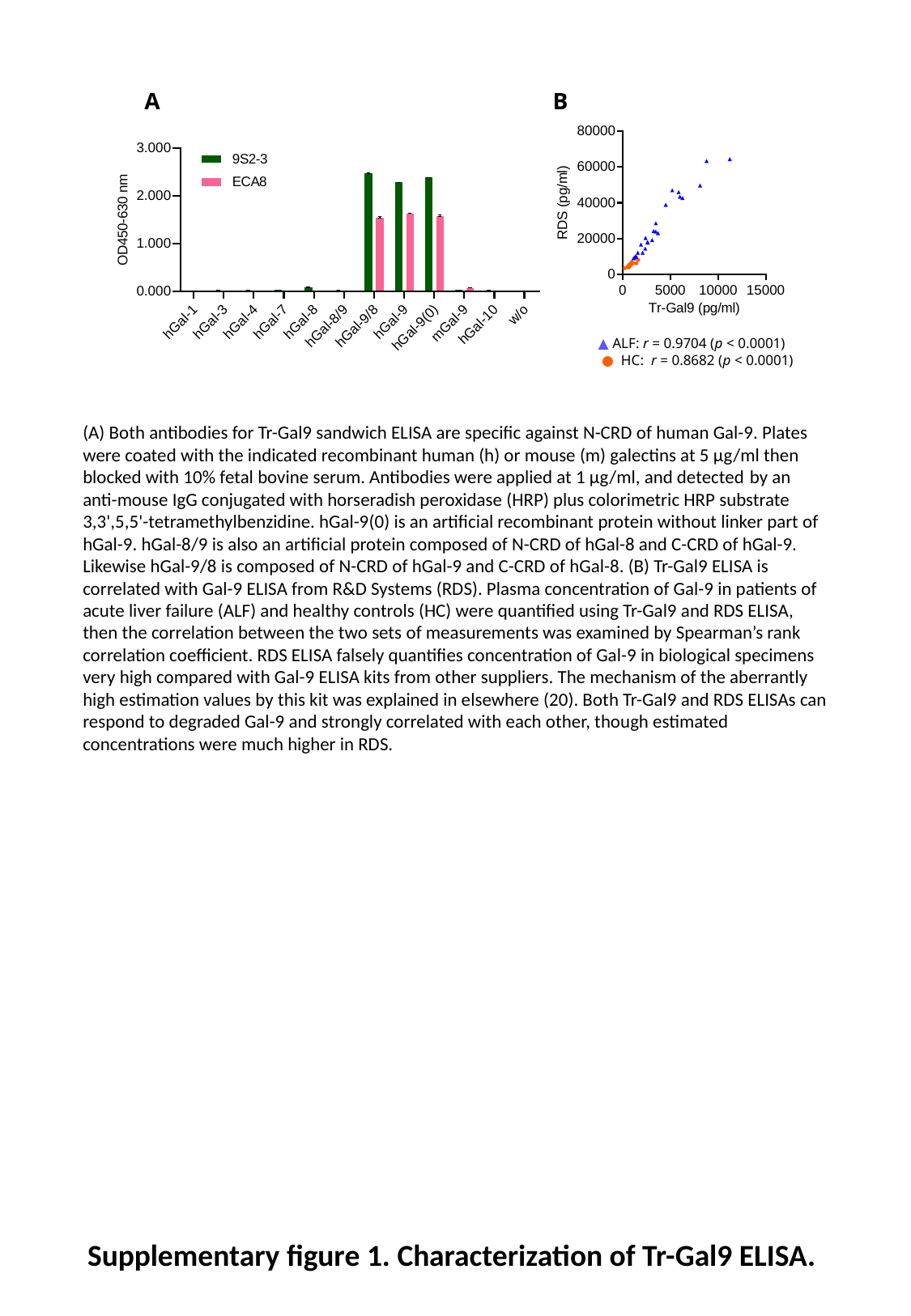

A B
▲ ALF: r = 0.9704 (p < 0.0001)
 ● HC: r = 0.8682 (p < 0.0001)
(A) Both antibodies for Tr-Gal9 sandwich ELISA are specific against N-CRD of human Gal-9. Plates were coated with the indicated recombinant human (h) or mouse (m) galectins at 5 µg/ml then blocked with 10% fetal bovine serum. Antibodies were applied at 1 µg/ml, and detected by an anti-mouse IgG conjugated with horseradish peroxidase (HRP) plus colorimetric HRP substrate 3,3',5,5'-tetramethylbenzidine. hGal-9(0) is an artificial recombinant protein without linker part of hGal-9. hGal-8/9 is also an artificial protein composed of N-CRD of hGal-8 and C-CRD of hGal-9. Likewise hGal-9/8 is composed of N-CRD of hGal-9 and C-CRD of hGal-8. (B) Tr-Gal9 ELISA is correlated with Gal-9 ELISA from R&D Systems (RDS). Plasma concentration of Gal-9 in patients of acute liver failure (ALF) and healthy controls (HC) were quantified using Tr-Gal9 and RDS ELISA, then the correlation between the two sets of measurements was examined by Spearman’s rank correlation coefficient. RDS ELISA falsely quantifies concentration of Gal-9 in biological specimens very high compared with Gal-9 ELISA kits from other suppliers. The mechanism of the aberrantly high estimation values by this kit was explained in elsewhere (20). Both Tr-Gal9 and RDS ELISAs can respond to degraded Gal-9 and strongly correlated with each other, though estimated concentrations were much higher in RDS.
Supplementary figure 1. Characterization of Tr-Gal9 ELISA.

## Slide 2
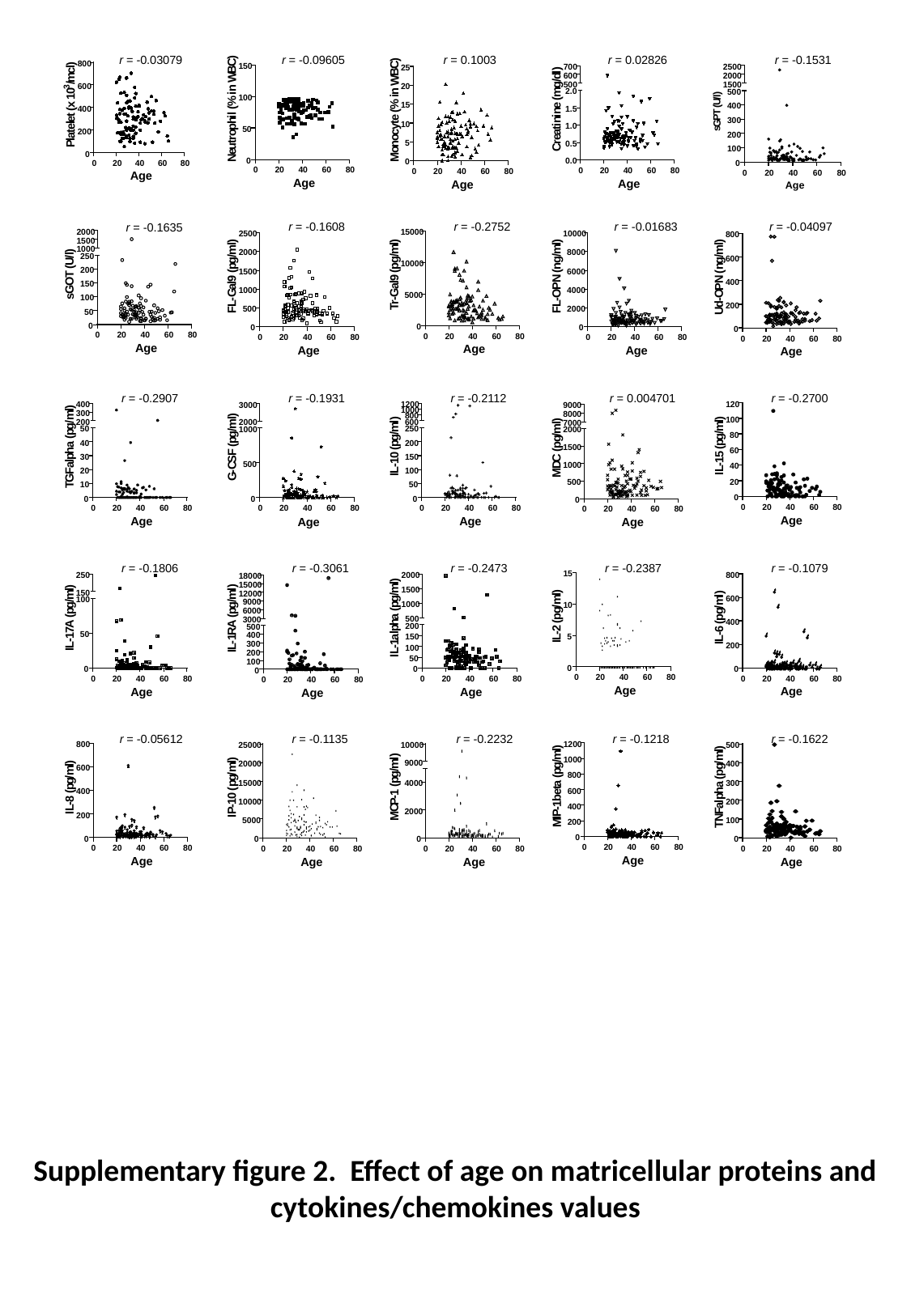

r = -0.03079
)
800
600
400
200
0
0
20
40
60
80
l
c
m
/
3
0
1
x
(
t
e
l
e
t
a
l
P
Age
)
r = -0.09605
C
150
100
50
0
0
20
40
60
80
B
W
n
i
%
(
l
i
h
p
o
r
t
u
e
N
Age
r = 0.1003
)
C
25
20
15
10
5
0
0
20
40
60
80
B
W
n
i
%
(
e
t
y
c
o
n
o
M
Age
r = 0.02826
700
600
500
2.0
1.5
1.0
0.5
0.0
0
20
40
60
80
)
l
d
/
g
m
(
e
n
i
n
i
t
a
e
r
C
Age
r = -0.1531
2500
2000
1500
500
400
300
200
100
0
0
20
40
60
80
Age
)
l
/
U
(
T
P
G
s
r = -0.1608
2500
)
l
m
2000
/
g
p
(
1500
9
l
a
1000
G
-
L
F
500
0
0
20
40
60
80
Age
r = -0.2752
15000
)
l
m
/
g
10000
p
(
9
l
a
G
5000
-
r
T
0
0
20
40
60
80
Age
r = -0.01683
10000
)
l
m
8000
/
g
n
(
6000
N
P
4000
O
-
L
F
2000
0
0
20
40
60
80
Age
r = -0.04097
800
)
l
m
/
g
600
n
(
N
400
P
O
-
d
200
U
0
0
20
40
60
80
Age
r = -0.1635
2000
1500
)
1000
l
/
250
U
(
200
T
O
150
G
s
100
50
0
0
20
40
60
80
Age
r = -0.1931
3000
2000
1000
F
S
C
500
-
G
0
0
20
40
60
80
Age
)
l
m
/
g
p
(
r = 0.004701
9000
8000
7000
2000
1500
1000
500
0
0
20
40
60
80
)
l
m
/
g
p
(
C
D
M
Age
r = -0.2907
400
300
200
50
40
30
20
10
0
0
20
40
60
80
)
l
m
/
g
p
(
a
h
p
l
a
F
G
T
Age
r = -0.2112
1200
1000
800
600
250
200
150
100
50
0
0
20
40
60
80
)
l
m
/
g
p
(
0
1
-
L
I
Age
r = -0.2700
120
100
80
60
40
20
0
0
20
40
60
80
)
l
m
/
g
p
(
5
1
-
L
I
Age
r = -0.3061
18000
15000
12000
9000
6000
3000
500
400
300
200
100
0
0
20
40
60
80
)
l
m
/
g
p
(
A
R
1
-
L
I
Age
r = -0.1079
800
)
l
600
m
/
g
p
(
400
6
-
L
I
200
0
0
20
40
60
80
Age
r = -0.1806
250
150
100
50
0
0
20
40
60
80
)
l
m
/
g
p
(
A
7
1
-
L
I
Age
r = -0.2473
2000
1500
1000
500
200
150
100
50
0
0
20
40
60
80
)
l
m
/
g
p
(
a
h
p
l
a
1
-
L
I
Age
r = -0.2387
15
)
l
m
/
10
g
p
(
2
-
L
5
I
0
0
20
40
60
80
Age
r = -0.05612
800
)
l
600
m
/
g
p
(
400
8
-
L
I
200
0
0
20
40
60
80
Age
r = -0.1218
1200
1000
800
600
400
200
0
0
20
40
60
80
)
l
m
/
g
p
(
a
t
e
b
1
-
P
I
M
Age
r = -0.1135
25000
)
l
20000
m
/
g
p
15000
(
0
1
10000
-
P
I
5000
0
0
20
40
60
80
Age
r = -0.2232
10000
)
l
m
9000
/
g
p
(
4000
1
-
P
C
2000
M
0
0
20
40
60
80
Age
r = -0.1622
500
400
300
200
100
0
0
20
40
60
80
)
l
m
/
g
p
(
a
h
p
l
a
F
N
T
Age
Supplementary figure 2. Effect of age on matricellular proteins and cytokines/chemokines values

## Slide 3
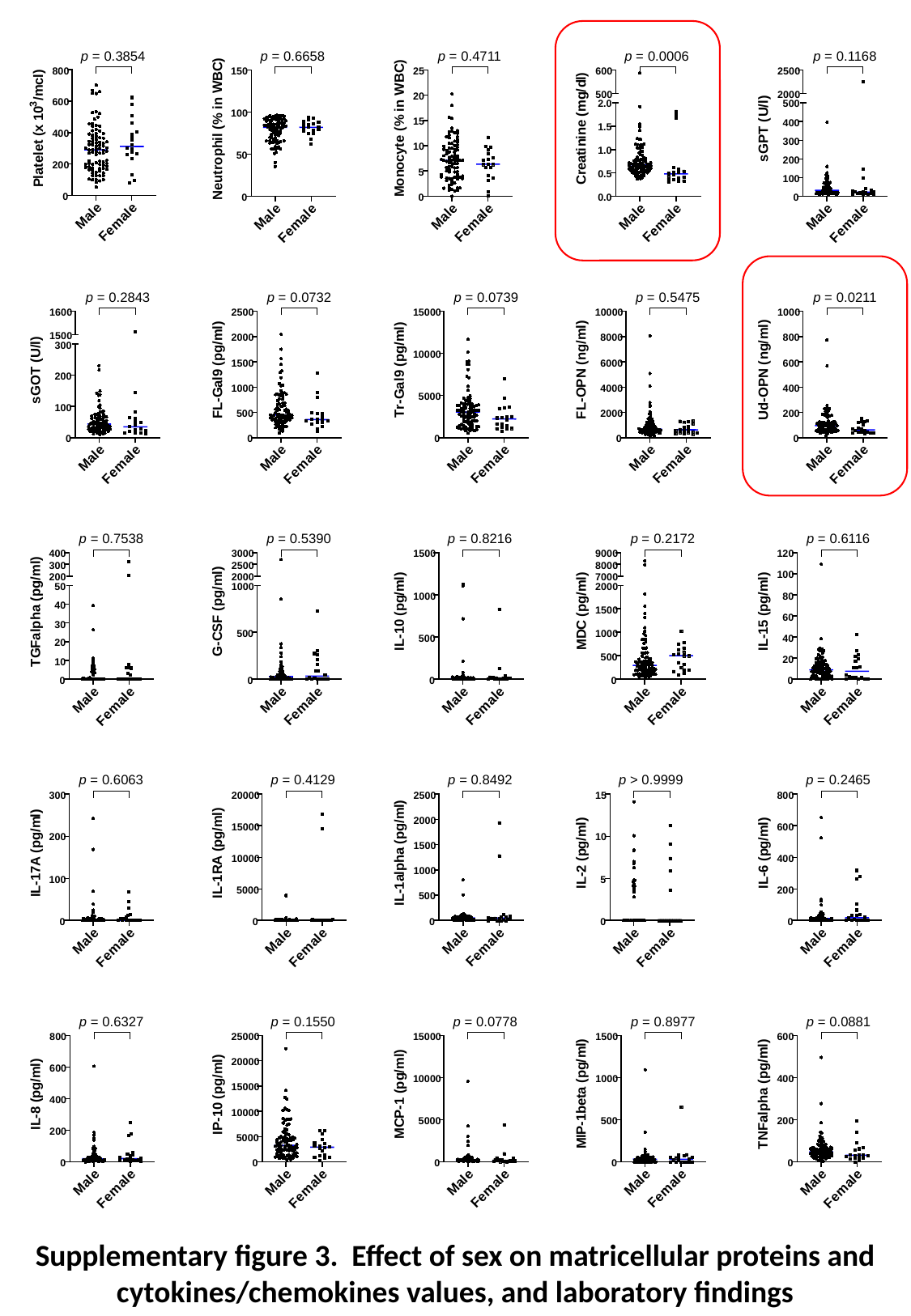

p = 0.3854
)
800
600
400
200
0
e
e
l
l
a
a
M
m
e
F
l
c
m
/
3
0
1
x
(
t
e
l
e
t
a
l
P
p = 0.6658
)
C
150
100
50
0
e
e
l
l
a
a
M
m
e
F
B
W
n
i
%
(
l
i
h
p
o
r
t
u
e
N
p = 0.4711
)
C
25
20
15
10
5
0
e
e
l
l
a
a
M
m
e
F
B
W
n
i
%
(
e
t
y
c
o
n
o
M
p = 0.0006
)
600
500
2.0
1.5
1.0
0.5
0.0
e
e
l
l
a
a
M
m
e
F
l
d
/
g
m
(
e
n
i
n
i
t
a
e
r
C
p = 0.1168
2500
2000
500
400
300
200
100
0
e
e
l
l
a
a
M
m
e
F
)
l
/
U
(
T
P
G
s
p = 0.2843
1600
1500
300
200
100
0
e
e
l
l
a
a
M
m
e
F
)
l
/
U
(
T
O
G
s
p = 0.0732
2500
2000
1500
1000
500
0
e
e
l
l
a
a
M
m
e
F
)
l
m
/
g
p
(
9
l
a
G
-
L
F
p = 0.0739
15000
10000
5000
0
e
e
l
l
a
a
M
m
e
F
)
l
m
/
g
p
(
9
l
a
G
-
r
T
p = 0.5475
10000
8000
6000
4000
2000
0
e
e
l
l
a
a
M
m
e
F
)
l
m
/
g
n
(
N
P
O
-
L
F
p = 0.0211
1000
800
600
400
200
0
e
e
l
l
a
a
M
m
e
F
)
l
m
/
g
n
(
N
P
O
-
d
U
p = 0.7538
400
300
200
50
40
30
20
T
10
0
e
e
l
l
a
a
M
m
e
F
)
l
m
/
g
p
(
a
h
p
l
a
F
G
p = 0.5390
3000
2500
2000
1000
500
0
e
e
l
l
a
a
M
m
e
F
)
l
m
/
g
p
(
F
S
C
-
G
p = 0.8216
1500
1000
p
(
0
1
-
500
L
I
0
e
e
l
l
a
a
M
m
e
F
)
l
m
/
g
p = 0.2172
9000
8000
7000
2000
1500
1000
500
0
e
e
l
l
a
a
M
m
e
F
)
l
m
/
g
p
(
C
D
M
p = 0.6116
120
100
80
60
40
20
0
e
e
l
l
a
a
M
m
e
F
)
l
m
/
g
p
(
5
1
-
L
I
p = 0.6063
300
200
(
A
7
1
-
100
L
I
0
e
e
l
l
a
a
M
m
e
F
)
l
m
/
g
p
p = 0.4129
20000
)
l
m
/
15000
g
p
(
A
10000
R
1
-
L
5000
I
0
e
e
l
l
a
a
M
m
e
F
p = 0.8492
2500
2000
1500
1000
500
0
e
e
l
l
a
a
M
m
e
F
)
l
m
/
g
p
(
a
h
p
l
a
1
-
L
I
p > 0.9999
15
)
l
m
/
10
g
p
(
2
-
L
5
I
0
e
e
l
l
a
a
M
m
e
F
p = 0.2465
800
600
400
200
0
e
e
l
l
a
a
M
m
e
F
)
l
m
/
g
p
(
6
-
L
I
p = 0.6327
800
600
400
200
0
e
e
l
l
a
a
M
m
e
F
)
l
m
/
g
p
(
8
-
L
I
p = 0.1550
25000
20000
15000
10000
5000
0
e
e
l
l
a
a
M
m
e
F
)
l
m
/
g
p
(
0
1
-
P
I
p = 0.0778
15000
10000
5000
0
e
e
l
l
a
a
M
m
e
F
)
l
m
/
g
p
(
1
-
P
C
M
p = 0.8977
1500
1000
500
0
e
e
l
l
a
a
M
m
e
F
)
l
m
/
g
p
(
a
t
e
b
1
-
P
I
M
p = 0.0881
600
400
200
0
e
e
l
l
a
a
M
m
e
F
)
l
m
/
g
p
(
a
h
p
l
a
F
N
T
Supplementary figure 3. Effect of sex on matricellular proteins and cytokines/chemokines values, and laboratory findings
