# Supplementary material for: Plasma Levels of a Cleaved Form of Galectin-9 Are the Most Sensitive Biomarkers of Acquired Immune Deficiency Syndrome and Tuberculosis Coinfection
Source: Biomolecules. 2020 Oct 30;10(11):1495. doi: 10.3390/biom10111495 (PMC7693693; doi:10.3390/biom10111495)
Supplement: Supplementary file 1 [file biomolecules-10-01495-s001.zip › biomolecules-943414-SI/biomolecules-943414-supplementary.docx]

**Supplementary Table 1. Correlation analysis of matricellular proteins in AIDS patients**

| **AIDS** | **FL-OPN** | | **Ud-OPN** | | **FL-Gal9** | | **Tr-Gal9** | |
| --- | --- | --- | --- | --- | --- | --- | --- | --- |
|  | ***r*** | **significance** | ***r*** | **significance** | ***r*** | **significance** | ***r*** | **significance** |
| **FL-OPN** | 1 |  | 0.89 | < 0.0001 | 0.10 | 0.64 | 0.38 | 0.08 |
| **Ud-OPN** |  |  | 1 |  | 0.18 | 0.39 | 0.42 | 0.04 |
| **FL-Gal9** |  |  |  |  | 1 |  | 0.70 | 0.0001 |
| **Tr- Gal9** |  |  |  |  |  |  | 1 |  |

Yellow indicates high correlation *r* > 0.6, Green indicate moderate correlation 0.4 ≤ *r* ≤ 0.6

Blue indicates low correlation 0.2 ≤ *r* < 0.4.

**Supplementary Table 2. Correlation analysis of matricellular proteins inTB patients**

| **TB** | **FL-OPN** | | **Ud-OPN** | | **FL-Gal9** | | **Tr-Gal9** | |
| --- | --- | --- | --- | --- | --- | --- | --- | --- |
|  | ***r*** | **significance** | ***r*** | **significance** | ***r*** | **significance** | ***r*** | **significance** |
| **FL-OPN** | 1 |  | 0.56 | < 0.0001 | (0.12) | 0.40 | 0.36 | 0.01 |
| **Ud-OPN** |  |  | 1 |  | 0.02 | 0.92 | 0.32 | 0.03 |
| **FL-Gal9** |  |  |  |  | 1 |  | 0.56 | < 0.0001 |
| **Tr- Gal9** |  |  |  |  |  |  | 1 |  |

Yellow indicate high correlation *r* > 0.6, Green indicate moderate correlation 0.4 ≤ *r* ≤ 0.6

Blue indicate low correlation 0.2 ≤ *r* < 0.4, red numerical value: minus value.

**Supplementary Table 3. Correlation analysis of matricellular proteins in AIDS/TB patients**

| **AIDS/TB** | **FL-OPN** | | **Ud-OPN** | | **FL-Gal9** | | **Tr-Gal9** | |
| --- | --- | --- | --- | --- | --- | --- | --- | --- |
|  | ***r*** | **significance** | ***r*** | **significance** | ***r*** | **significance** | ***r*** | **significance** |
| **FL-OPN** | 1 |  | 0.69 | < 0.0001 | (0.23) | 0.20 | 0.34 | 0.05 |
| **Ud-OPN** |  |  | 1 |  | (0.11) | 0.54 | 0.15 | 0.41 |
| **FL-Gal9** |  |  |  |  | 1 |  | 0.41 | 0.02 |
| **Tr- Gal9** |  |  |  |  |  |  | 1 |  |

Yellow indicate high correlation *r* > 0.6, Green indicate moderate correlation 0.4 ≤ *r* ≤ 0.6

Blue indicate low correlation 0.2 ≤ *r* < 0.4, red numerical value: minus value.

.

**Supplementary Table 4. Diagnostic accuracy of molecules between AIDS and normal**

| **molecule** | **threshold** | **specificity** | **sensitivity** | **npv*** | **ppv**** | **youden** |
| --- | --- | --- | --- | --- | --- | --- |
| **FL-Gal9** | 233 | 0.926 | 0.958 | 0.962 | 0.920 | 1.884 |
| **Tr-Gal9** | 724 | 0.963 | 1.000 | 1.000 | 0.960 | 1.963 |
| **FL-OPN** | 248 | 1.000 | 0.957 | 0.968 | 1.000 | 1.957 |
| **Ud-OPN** | 43.5 | 1.000 | 1.000 | 1.000 | 1.000 | 2.000 |
| **IP-10** | 742 | 0.967 | 0.958 | 0.967 | 0.958 | 1.925 |
| **TNFα** | 22.5 | 0.933 | 0.917 | 0.933 | 0.917 | 1.850 |
| **IL-8** | 11.5 | 0.900 | 0.708 | 0.794 | 0.850 | 1.608 |
| **IL-10** | 6.50 | 0.900 | 0.750 | 0.818 | 0.857 | 1.650 |
| **MDC** | 287 | 0.933 | 0.667 | 0.778 | 0.889 | 1.600 |

***: negative predictive value. **; positive predictive value**

**Supplementary Table 5. Diagnostic accuracy of molecules between TB and normal**

| **molecule** | **threshold** | **specificity** | **sensitivity** | **npv** | **ppv** | **youden** |
| --- | --- | --- | --- | --- | --- | --- |
| **FL-Gal9** | 167 | 0.852 | 0.918 | 0.852 | 0.918 | 1.770 |
| **Tr-Gal9** | 165 | 0.889 | 0.980 | 0.960 | 0.941 | 1.868 |
| **FL-OPN** | 254 | 1.000 | 1.000 | 1.000 | 1.000 | 2.000 |
| **Ud-OPN** | 27.5 | 0.933 | 1.000 | 1.000 | 0.961 | 1.933 |
| **IL-2** | 0.50 | 0.867 | 0.837 | 0.765 | 0.911 | 1.703 |
| **TNFα** | 21.5 | 0.900 | 0.816 | 0.750 | 0.930 | 1.716 |
| **IL-8** | 10.5 | 0.867 | 0.816 | 0.743 | 0.909 | 1.683 |
| **IP-10** | 720 | 0.967 | 0.959 | 0.935 | 0.979 | 1.926 |

**Footnotes are the same as Supplementary Table 4.**

**Supplementary Table 6. Diagnostic accuracy of molecules between AIDS/TB and normal**

| **molecule** | **threshold** | **specificity** | **sensitivity** | **npv** | **ppv** | **youden** |
| --- | --- | --- | --- | --- | --- | --- |
| **FLGal9** | 252 | 0.963 | 1.000 | 1.000 | 0.971 | 1.963 |
| **Tr-Gal9** | 1640 | 1.000 | 1.000 | 1.000 | 1.000 | 2.000 |
| **FL-OPN** | 251 | 1.000 | 0.970 | 0.968 | 1.000 | 1.970 |
| **Ud-OPN** | 38.5 | 0.967 | 0.970 | 0.967 | 0.970 | 1.936 |
| **IL-1α** | 34.0 | 0.733 | 0.909 | 0.880 | 0.789 | 1.642 |
| **IL-10** | 6.50 | 0.900 | 0.848 | 0.844 | 0.903 | 1.748 |
| **IL-15** | 8.50 | 0.833 | 0.758 | 0.758 | 0.833 | 1.591 |
| **TNFα** | 23.5 | 0.967 | 0.939 | 0.935 | 0.969 | 1.906 |
| **IL-8** | 11.5 | 0.900 | 0.818 | 0.818 | 0.900 | 1.718 |
| **IP-10** | 701 | 0.967 | 1.000 | 1.000 | 0.971 | 1.967 |
| **MCP-1** | 212 | 0.767 | 0.848 | 0.821 | 0.800 | 1.615 |

**Footnotes are the same as Supplementary Table 4.**

**Supplementary Table 7. Accuracy of severity of each disease**

| **disease** | **molecule** | **threshold** | **specificity** | **sensitivity** | **npv** | **ppv** | **youden** |
| --- | --- | --- | --- | --- | --- | --- | --- |
| **AIDS** | Tr-Gal9 | 7068 | 0.905 | 1.000 | 1.000 | 0.600 | 1.905 |
|  | FL-Gal9 | 848 | 0.762 | 1.000 | 1.000 | 0.375 | 1.762 |
|  | IL-1RA | 57.5 | 0.857 | 1.000 | 1.000 | 0.500 | 1.857 |
|  | IL-17A | 6.00 | 0.905 | 1.000 | 1.000 | 0.600 | 1.905 |
|  | TGFα | 6.50 | 0.905 | 1.000 | 1.000 | 0.600 | 1.905 |
|  | IL-8 | 40.0 | 0.762 | 1.000 | 1.000 | 0.375 | 1.762 |
|  | MDC | 4452 | 1.000 | 0.667 | 0.955 | 1.000 | 1.667 |
|  | MIP-1α | 60.5 | 0.810 | 1.000 | 1.000 | 0.429 | 1.810 |
|  | | | | | | |  |
| **TB** | IL-6 | 15.5 | 0.698 | 1.000 | 1.000 | 0.316 | 1.698 |
|  | G-CSF | 24.5 | 0.651 | 1.000 | 1.000 | 0.286 | 1.651 |
|  | MCP-1 | 270 | 0.837 | 0.833 | 0.973 | 0.417 | 1.671 |
|  | | | | | | |  |
| **AIDS/TB** | MIP-1β | 66.5 | 0.964 | 0.800 | 0.964 | 0.800 | 1.764 |
